# The MERS-CoV N Protein Regulates Host Cytokinesis and Protein Translation via Interaction With EF1A

**DOI:** 10.3389/fmicb.2021.551602

**Published:** 2021-06-23

**Authors:** Lin Zhu, Ting Gao, Yangbo Fu, Xiujing Han, Junjie Yue, Yaoning Liu, Hainan Liu, Qincai Dong, Weihong Yang, Yong Hu, Yanwen Jin, Ping Li, Xuan Liu, Cheng Cao

**Affiliations:** ^1^Beijing Institute of Biotechnology, Army Institute of Military Medical Sciences, Academy of Military Sciences, Beijing, China; ^2^Institute of Physical Science and Information Technology, Anhui University, Hefei, China; ^3^Department of Clinical Laboratory, First Affiliated Hospital of Guangzhou Medical University, Guangzhou, China

**Keywords:** MERS-CoV, nucleocapsid protein, EF1A, cytokinesis, multiple nuclei, F-actin

## Abstract

Middle East respiratory syndrome coronavirus (MERS-CoV), a pathogen causing severe respiratory disease in humans that emerged in June 2012, is a novel beta coronavirus similar to severe acute respiratory syndrome coronavirus (SARS-CoV). In this study, immunoprecipitation and proximity ligation assays revealed that the nucleocapsid (N) protein of MERS-CoV interacted with human translation elongation factor 1A (EF1A), an essential component of the translation system with important roles in protein translation, cytokinesis, and filamentous actin (F-actin) bundling. The C-terminal motif (residues 359–363) of the N protein was the crucial domain involved in this interaction. The interaction between the MERS-CoV N protein and EF1A resulted in cytokinesis inhibition due to the formation of inactive F-actin bundles, as observed in an *in vitro* actin polymerization assay and in MERS-CoV-infected cells. Furthermore, the translation of a CoV-like reporter mRNA carrying the MERS-CoV 5′UTR was significantly potentiated by the N protein, indicating that a similar process may contribute to EF1A-associated viral protein translation. This study highlights the crucial role of EF1A in MERS-CoV infection and provides new insights into the pathogenesis of coronavirus infections.

## Introduction

The coronaviruses are a large family of viruses that are common pathogens in animals. The single-stranded nucleic acid genome carried by coronaviruses is among the largest known mature RNA viral genomes. Like other RNA viruses, coronaviruses are prone to mutation, by which they can adapt to new hosts, such as humans. The coronaviruses that infect humans are primarily respiratory pathogens, although they are universally associated with diarrhea as well as the common cold (Lai and Cavanagh, 1997). Over the last decade, three zoonotic coronaviruses, severe acute respiratory syndrome (SARS) coronavirus (SARS-CoV), SARS coronavirus 2 (SARS-CoV-2), and Middle East respiratory syndrome (MERS) coronavirus (MERS-CoV), have been identified to cause severe acute lower respiratory tract diseases with high mortality in humans (Ksiazek et al., 2003; Xu et al., 2020).

Middle East respiratory syndrome coronavirus, which was first isolated from a patient who died from a severe respiratory illness in September 2012 in Jeddah, Saudi Arabia, is an emerging member of the genus Betacoronavirus and has been identified as the etiological agent that causes MERS (Zaki et al., 2012) with a mortality rate as high as 34.4% (Peiris et al., 2004). MERS-CoV is an enveloped virus with a single positive-sense RNA genome (size: ∼30 kb) that encodes spike (S) protein, small envelope (E) protein, membrane (M) protein, and nucleocapsid (N) protein. The MERS-CoV N protein is a ∼40 kDa protein organized into two main domains, the N-terminal domain (NTD) and the C-terminal domain (CTD) separated by a disordered region (called the LKR). The N protein binds to RNA to form a ribonucleoprotein complex that participates in viral replication and assembly (Papageorgiou et al., 2016; Li et al., 2019; Nguyen et al., 2019). Both the NTD and CTD of the MERS-CoV N protein are responsible for RNA binding, while CTD is responsible for dimerization of N monomers (Papageorgiou et al., 2016). The N proteins of coronaviruses, including transmissible gastroenteritis coronavirus (TGEV), infectious bronchitis virus (IBV), and SARS-CoV, can induce cells to become multinucleated or to undergo apoptosis (Surjit et al., 2004; Li et al., 2007; Yang et al., 2010). Similarly, MERS-CoV has also been reported to regulate protein translation (Zhou et al., 2015). However, the exact molecular mechanism is unclear.

The translation elongation factors (EFs) are a set of proteins responsible for translation elongation during protein synthesis (Fessenden et al., 1963). There are three EFs in eukaryotic cells: EF-1, EF-2, and EF-3. Elongation factor 1A (also known as EF1α), which is equivalent to the prokaryotic elongation factor EF-Tu, is one of the four subunits of EF-1 (Ejiri, 2002). EF1A is not only a major translational factor in mammalian cells but is also involved in substance transport to the nucleus, apoptotic signal transduction, virion packaging (Cimarelli and Luban, 1999), and cytoskeletal organization (Gross and Kinzy, 2005; Bunai et al., 2006).

In this study, we found that the N protein of MERS-CoV can interact with EF1A, blocking its association with actin and promoting protein synthesis. As a result, cytokinesis and cell proliferation are inhibited and multiple nuclei formation occurs in cells expressing the MERS-CoV N protein, similar to the case in cells expressing the SARS-CoV N protein (Zhou et al., 2008). These findings might explain the lymphopenia that occurs in MERS patients (Arabi et al., 2015).

## Materials and Methods

### Cell Culture, Vectors, and Viruses

HEK293T and HeLa cells were obtained from the American Type Culture Collection (ATCC) and cultured in Dulbecco’s modified Eagle’s medium (DMEM) (Gibco) containing 10% fetal bovine serum (Gibco), 100 U/ml of penicillin, and 100 U/ml of streptomycin. The cells were transfected with plasmid DNA using Lipofectamine 2000 (Invitrogen) according to the manufacturer’s protocol.

GFP-tagged SARS-CoV N (GFP-SARS N), GFP-tagged MERS-CoV N (GFP-MERS N), Flag-tagged MERS-CoV N (Flag-MERS N), and MERS N mutants were expressed by cloning the genes into pEGFP-C1 (Clontech). EF1A-Flag (GenBank: EF362804.1) and EF1A mutants were constructed by cloning the gene fragments into a pcDNA3.1-based Flag-vector (Invitrogen). All of the above constructs were validated by Sanger DNA sequencing prior to use.

An adenovirus expressing the MERS-CoV N protein (Ad-MERS N) was provided by BAC Biological Technology Co., Ltd. To express MERS-CoV N in cells, cells were inoculated with Ad-MERS N at a multiplicity of infection (MOI) of 10. The EMC-2012 strain of MERS-CoV was kindly provided by Yusen Zhou and Guangyu Zhao (Beijing Institute of Microbiology and Epidemiology, China). Vero E6 cells were obtained from the ATCC, cultured in DMEM containing 10% fetal bovine serum, and inoculated with MERS-CoV (passage 2) at an MOI of 0.1. The infectious virus work was performed in a BSL-3 laboratory at the Beijing Institute of Microbiology and Epidemiology.

### Antibodies and Reagents

A horseradish peroxidase (HRP)-labeled antibody against EF1A was obtained from Bioss. An antibody against Flag (HRP), beads coupled with Flag antibodies, and antibodies against human actin and rabbit IgG (HRP) were obtained from Sigma-Aldrich. Protein A/G beads, anti-rabbit IgG beads, and an antibody against EF1A were purchased from Santa Cruz Biotechnology. An antibody against GFP (HRP) was purchased from Proteintech. Antibodies against MERS-CoV N were obtained from Sino Biological Inc. Actin protein from rabbit skeletal muscle, a Flag peptide, DAPI dye, latrunculin B, and jasplakinolide were obtained from Sigma-Aldrich. Phalloidin labeled with TRITC was purchased from Yeasen. A rabbit reticulocyte lysis system and RNase inhibitor were obtained from Promega.

### Immunoprecipitation and Immunoblotting

Cells were collected by centrifugation at 2,000 rpm and lysed using lysis buffer (50 mM Tris–HCl pH 7.4, 150 mM NaCl, and 1% Nonidet P-40 with Roch’s protease inhibitor). The supernatant of the lysate was separated by centrifugation at 16,000 × *g* at 4°C. Agarose beads labeled with Flag or protein A/G beads combined with an EF1A antibody were added into the supernatant and incubated for 6 h at 4°C. The beads were then subjected to denaturing polyacrylamide gel electrophoresis (SDS-PAGE) in electrophoresis buffer (25 mM Tris base, 250 mM glycine, and 0.1% SDS) and washed four times using lysis buffer without protease inhibitor. After electrophoresis, the proteins contained in the polyacrylamide gel were transferred onto a PVDF immunoblotting membrane in transfer buffer (24 mM Tris base, 192 mM glycine, and 20% methanol) for 1.5 h at 18 volts. The PVDF membrane was blocked with 5% skim milk for 1 h at room temperature and then incubated with the indicated primary antibodies overnight at 4°C before being incubated with the relevant secondary antibodies for 40 min at room temperature. The PVDF membrane was washed three times using TBST [20 mM Tris–HCl (pH 7.4), 150 mM NaCl, 0.05% Tween-20] after incubation. The antigen-antibody complexes were visualized using an enhanced chemiluminescence detection system (Amersham Biosciences/GE Healthcare, Buckinghamshire, United Kingdom).

### Immunofluorescence

Cells were rinsed three times with PBS (137 mM NaCl, 2.7 mM KCl, 10 mM Na_2_HPO_4_, 1.8 mM KH_2_PO_4_), fixed in 4% formaldehyde in PBS at room temperature for 30 min, washed three times with PBS, permeabilized with 0.2% Triton X-100 in PBS at room temperature for 5 min, and washed twice with PBS. Filamentous actin (F-actin) was dyed with rhodamine-labeled phalloidin (to mark the cytoskeleton) at room temperature for 20 min, and the cells were then rinsed four times with PBS. The nuclei were stained with DAPI. Images were acquired using a Zeiss LSM 800 Meta confocal microscope.

### *In situ* Proximity Ligation Assay

A Duolink *in situ* proximity ligation assay (PLA) (Sigma-Aldrich) was used to detect interactions between EF1A and the MERS-CoV N protein in MERS-CoV-infected cells. In brief, Vero E6 cells plated on glass coverslips were infected with MERS-CoV at an MOI of 0.1. At 48 h post infection, the cells were fixed with 4% formaldehyde for 30 min and then rinsed with PBS. Next, the cells on glass coverslips were permeabilized with 0.3% Triton X-100 in PBS for 15 min. After the cells were blocked with the blocking buffer, they were incubated with antibodies against EF1A (Santa Cruz Biotechnology) and MERS-CoV N (Sino Biological Inc.) according to the manufacturer’s instructions for the PLA. F-actin and nuclei were stained as described above. Red fluorescence was generated from a DNA amplification-based reporter system involving reporters combined with oligonucleotide-labeled secondary antibodies and detected with a Zeiss LSM 800 Meta confocal microscope (Carl Zeiss) at a magnification of 63×.

### Purification of the MERS-CoV N Protein

As described previously (Zhou et al., 2008), the prokaryotic expression plasmid pET-22b(+)-MERS-CoV N was transformed into the expression strain BL21(DE3). After induction with 1 mM isopropyl-D-thiogalactopyranoside (IPTG) overnight at 30°C, the bacteria were harvested by centrifugation and lysed in buffer including 25 mM Na_2_HPO_4_, 25 mM NaH_2_PO_4_, 1 mM EDTA (pH 8.0), and protease inhibitor by ultrasonication. The soluble N protein in the ultrasonicated mixture was purified using strong cation exchange chromatography with SP-Sepharose Fast Flow resin followed by Superdex 200 gel filtration (GE Healthcare). The *E. coli* transformed with the vector plasmid pET-22b(+) were processed in the same way, and the final eluate was used as a negative control for purified N protein.

### Preparation of EF1A-Flag Protein

HEK293T cells expressing EF1A-Flag or Flag control constructs were harvested by centrifugation and lysed with cell lysis buffer as described above. The EF1A-Flag protein was precipitated with Flag-labeled agarose beads overnight at 4°C and further eluted with Flag peptide.

### Flow Cytometry

HeLa cells were rinsed three times with PBS and fixed in 70% ethanol overnight at −20°C, following centrifugation at 2,000 × *g* for 5 min. Then, the cells were washed twice with PBS and digested with 20 μg/ml RNase A for 20 min at room temperature. Finally, samples were stained with 50 μg/ml of propidium iodide at room temperature for 15 min, and the propidium iodide intensity was analyzed by a flow cytometer (Millipore ImageStreamX MarkII).

### *In vitro* Translation Assay

A recombinant N protein or a control protein was incubated with 17 ml of rabbit reticulocyte lysate (RRL; Promega). One hour later, 200 ng of firefly luciferase-encoding mRNA and amino acids were added to the mixture, which was incubated for another 1.5 h at 30°C. Luciferase activity was measured with a TD-20/20 luminometer.

### Reporter mRNAs Translation Assay

Reporter RNAs carrying CoV 5′UTRs were designed according to the MERS-CoV subgenomic (sg) mRNA sequence or the 5′UTR of β-actin as a control and synthesized with an mMESSAGE mMACHINE T7 Ultra Kit (Thermo). HEK293T cells were transfected with *in vitro* RNA transcripts using TransIT mRNA (Mirus). The levels of mCherry expression were detected 24 h after RNA transfection using a multilabel plate reader. Luciferase activity was measured with a TD-20/20 luminometer.

### *In vitro* Actin Polymerization Assay

This assay was performed as previously reported (Bunai et al., 2006), with some modifications. Rabbit skeletal muscle actin was solubilized in buffer (20 mM HEPES, 2 mM MgCl_2_, 2 mM EGTA, 1 mM DTT, 1 mM ATP, 0.25 mM GDP, and 1 mM PMSF), and residual actin polymers were removed by ultracentrifugation at 150,000 × *g* for 30 min at 4°C. Then, the collected rabbit G-actin (0.5 μM) was mixed with purified proteins, including the MERS-CoV N protein, the EF1A-Flag protein, and control proteins such as Flag peptide, in a total volume of 100 μl. The mixture was incubated at 4°C overnight in the presence or absence of 1 μM latrunculin B as a negative control or 1 μM jasplakinolide as a positive control. The actin polymers in the mixture were enriched by centrifugation at 50,000 × *g* for 2 min at 4°C, and the supernatants and precipitates were analyzed by immunoblotting with an actin antibody.

### Negative Staining and Electron Microscopy

The actin polymer-containing mixture prepared above was mounted on copper grids at room temperature and negatively stained with 4% uranyl acetate. The copper grids were observed under a Hitachi H-7650 transmission electron microscope at an accelerating voltage of 80 kV.

### Statistical Analysis

The results presented as the mean ± SD. Statistical analyses were performed using Prism (version 6.0, GraphPad Software). The difference between two groups was analyzed by the two-tailed Student’s *t*-test, and was considered statistically significant at ^∗^*p* < 0.05, ^∗∗^*p* < 0.01, and ^∗∗∗^*p* < 0.001.

## Results

### The MERS-CoV N Protein Associates With EF1A

**FIGURE 1:**
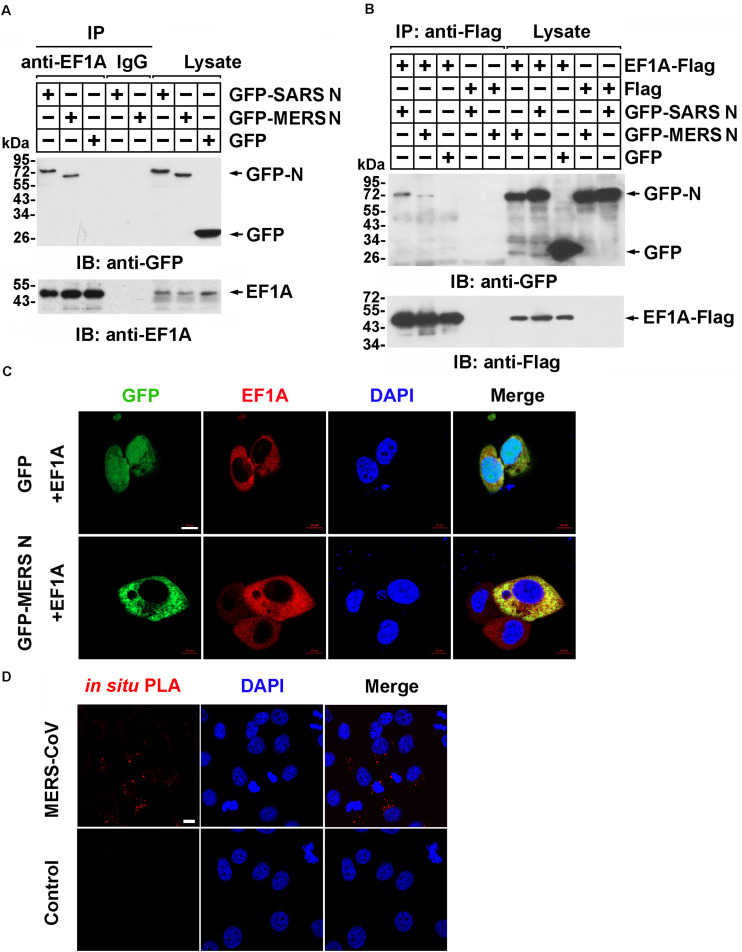
The MERS-CoV N protein associates with EF1A. **(A)** HEK293T cells transfected with the indicated plasmids. After 48 h, the cell were lysed and incubated with an EF1A antibody or normal rabbit IgG as a control and protein A/G agarose beads. The immunoprecipitates were detected by immunoblotting with anti-GFP and anti-EF1A antibodies. **(B)** HEK293T cells were transfected with the indicated plasmids. After 48 h, the cell were lysed and incubated with anti-Flag beads. Then, the immunoprecipitates were analyzed by immunoblotting with anti-GFP and anti-Flag antibodies. **(C)** HeLa cells were cotransfected with GFP-MERS N (or GFP) and EF1A-Flag expression plasmids. After 48 h, cells were fixed and incubated with an anti-EF1A antibody to detect the colocalization of MERS N (green) with EF1A (red) in HeLa cells via confocal microscopy. Nuclei were stained with DAPI (blue). Scale bar, 10 μm. **(D)** Vero E6 cells were infected with MERS-CoV (MOI = 0.1) and subjected to an *in situ* PLA. The red dots indicate the interaction signals, and nuclei are stained with DAPI (blue). Scale bar, 10 μm.

Our previous study demonstrated that human EF1A associates with the SARS-CoV N protein (Zhou et al., 2008), which shares 51% identity with the MERS-CoV N protein predicted by SWISS (Supplementary Figure 1). To investigate whether the N protein of MERS-CoV interacts with EF1A, lysates from HEK293T cells expressing GFP-SARS-CoV N protein, GFP-MERS-CoV N protein, or GFP were incubated with an EF1A antibody, and immunoprecipitation was performed with protein A/G agarose beads. The immunoprecipitates were separated by SDS-PAGE and analyzed by immunoblotting with anti-EF1A and anti-GFP antibodies. Similar to the findings for the SARS-CoV N protein, an association between GFP-MERS-CoV N and endogenous EF1A was observed (Figure 1A). Then, Flag-tagged EF1A was expressed in HEK293T cells, and an interaction between overexpressed EF1A-Flag and GFP-MERS N was also observed (Figure 1B). Further, HeLa cells co-expressing GFP-MERS N (or GFP) and EF1A-Flag were immunostained with anti-EF1A antibodies, and an obvious cytoplasmic colocalization of MERS-CoV N protein and EF1A was observed (Figure 1C). The interaction of EF1A with the N protein in the cytoplasm (red puncta) of MERS-CoV-infected cells was also demonstrated by *in situ* PLA (Figure 1D).

### The C-Terminus of the MERS-CoV N Protein Interacts With Domain III of EF1A

**FIGURE 2:**
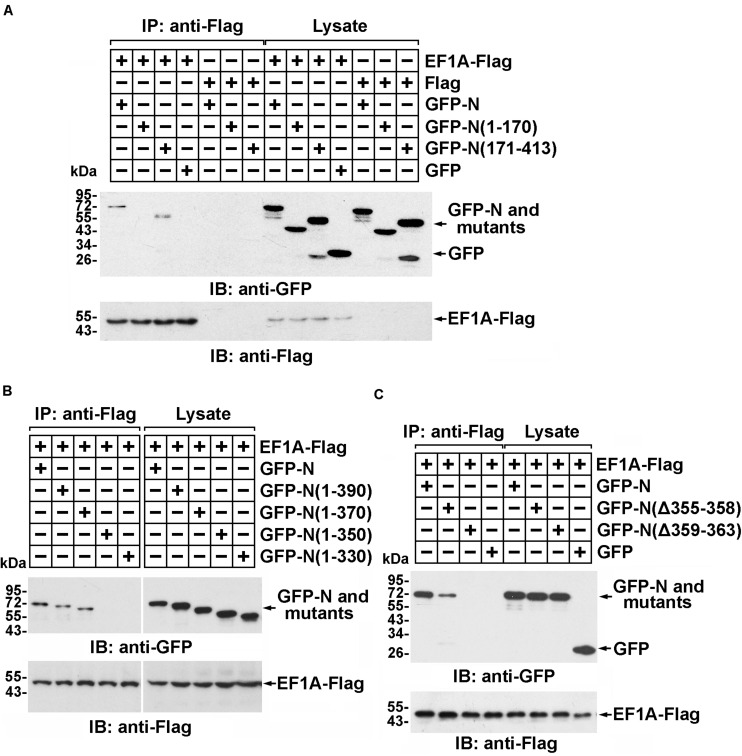
Residues 359–363 of the MERS-CoV N protein and the C-terminal motif of EF1A are the crucial domains involved in the MERS-CoV N-EF1A interaction. **(A,B)** The C-terminus of the MERS-CoV N protein contributes to the association with EF1A. HEK293T cells were transfected with EF1A-Flag and GFP-N, mutant constructs, or GFP (negative control vectors). Forty-eight hours after transfection, the cells were lysed and incubated with anti-Flag beads. Then, the immunoprecipitates were detected by immunoblotting with anti-GFP and anti-Flag antibodies. **(C)** Anti-Flag immunoprecipitates prepared from lysates of HEK293T cells expressing the GFP-MERS-CoV N protein and EF1A-Flag or mutants were assayed by immunoblotting. The GFP-tagged N protein, Flag-tagged EF1A, and mutants were detected with anti-GFP and anti-Flag antibodies.

The interaction between the SARS-CoV N protein and EF1A relies on the C-terminal self-association domain of the SARS-CoV N protein (Zhou et al., 2008). Consistent with this finding, immunoprecipitation followed by immunoblot assays showed that amino acid residues 171–413 of the MERS-CoV N protein, rather than amino acid residues 1–170, associated with EF1A (Figure 2A and Supplementary Figure 2A). To further determine the key region of the MERS-CoV N protein involved in the EF1A interaction, a series of N protein truncation mutants were constructed. The immunoprecipitation assays showed that amino acids 1–370 and 1–390 of the N protein, but not amino acids 1–350, or the other truncated mutants, could be associated with EF1A (Figure 2B). Therefore, the key motif of the MERS-CoV N protein responsible for EF1A association was determined to be located at amino acids 350–370. Accordingly, a key component of the interacting region was mapped to a tract of five residues (amino acids 359–363) at the downstream end of the CTD of the N protein in an α-helix (Figure 2C and Supplementary Figures 2B,C).

**FIGURE 3:**
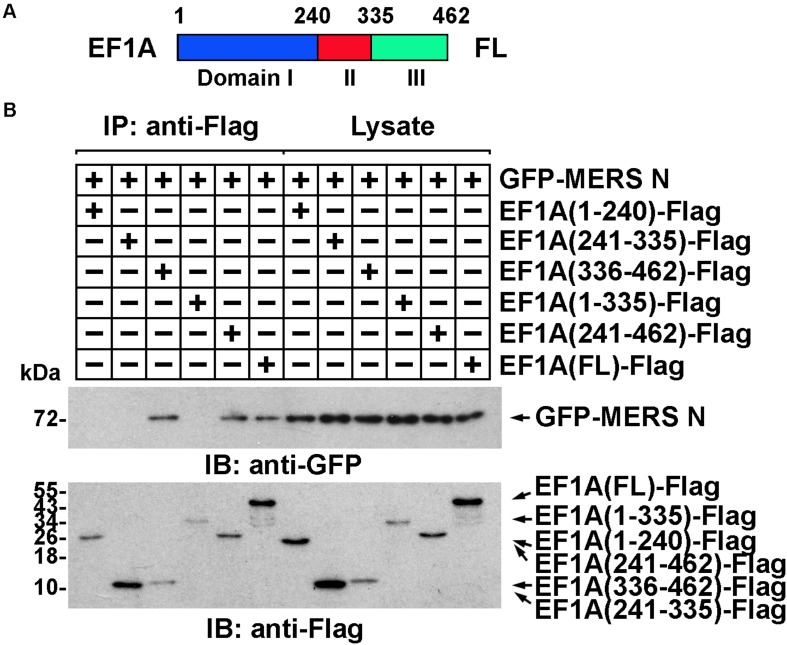
The interaction between EF1A and the MERS-CoV N protein primarily depends on the third (C-terminal) domain of EF1A. **(A)** Schematic representation of eEF1A protein domains. **(B)** Anti-Flag immunoprecipitates prepared from lysates of HEK293T cells expressing the GFP-MERS-CoV N protein and EF1A-Flag or mutants were assayed by immunoblotting. The GFP-tagged N protein, Flag-tagged EF1A, and mutants were detected with the indicated antibodies.

The domain of EF1A involved in the interaction was similarly determined. Truncated EF1A mutants were constructed based on the three structural domains of EF1A: I, II, and III (Figure 3A) (Hasler et al., 2011). An immunoprecipitation assay demonstrated that the interaction between EF1A and the MERS-CoV N protein primarily depended on the β-sheet motif in domain III of EF1A (in the C-terminus) (Figure 3B and Supplementary Figure 2D), which was also confirmed to be involved in actin binding (Kurasawa et al., 1996). This finding indicates that the MERS-CoV N protein might interfere with the association between EF1A and actin by competitively occupying the CTD of EF1A.

### The MERS-CoV N Protein Blocks the EF1A-Actin Interaction and Disturbs Actin Bundling

**FIGURE 4:**
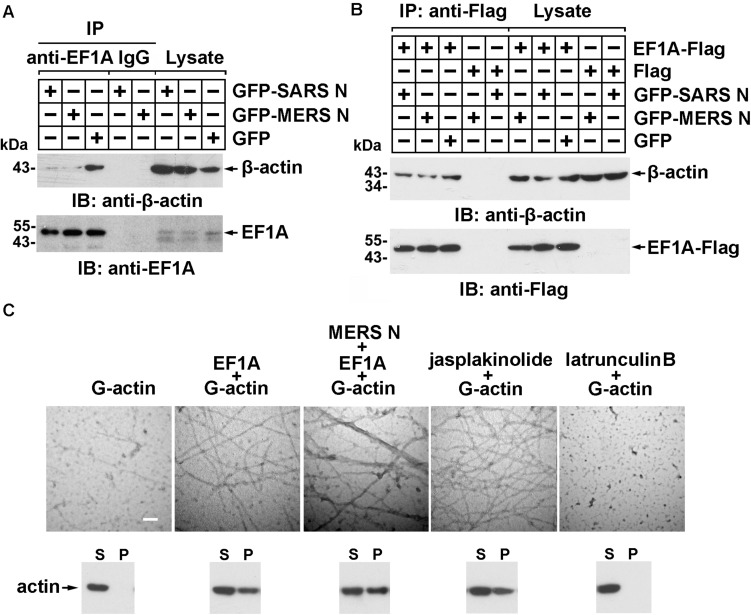
The MERS-CoV N protein blocks the interaction between EF1A and actin and disturbs actin bundling. **(A)** Lysates prepared from HEK293T cells transfected with plasmids expressing GFP-SARS-CoV N, GFP-MERS-CoV N, or GFP protein were incubated with an EF1A antibody and protein A/G agarose beads. The immunoprecipitates were detected by immunoblotting with the indicated antibodies. **(B)** Lysates prepared from HEK293T cells cotransfected with plasmids expressing GFP-tagged N proteins and Flag-tagged EF1A protein were incubated with anti-FLAG M2 magnetic beads. The immunoprecipitates were detected by immunoblotting with the indicated antibodies. **(C)** An *in vitro* actin polymerization assay was performed. G-actin (0.5 μM) was incubated with the indicated purified proteins at 4°C overnight. The products of the actin polymerization assay were mounted on copper grids and subjected to negative staining. The samples were observed with a Hitachi H-7650 transmission electron microscope at an accelerating voltage of 80 kV. The scale bars represent 100 nm. The polymerized actin (pellet, P) and monomeric actin (supernatant, S) in each product above were also separated by centrifugation and analyzed using immunoblotting. Jasplakinolide (1 μM) and latrunculin B (1 μM) were used as positive and negative controls, respectively.

More than 60% of total cellular EF1A is bound to F-actin (Gross and Kinzy, 2005), which promotes F-actin bundling. To assess whether the MERS-CoV N protein affects the interaction between EF1A and actin, lysates from cells expressing GFP-N protein or GFP were subjected to anti-EF1A antibody and protein A/G bead immunoprecipitation and detected by immunoblotting with anti-GFP antibody. The results showed that both the MERS-CoV N protein and the SARS-CoV N protein blocked the binding of endogenous EF1A to actin (Figure 4A), but this effect could be partially rescued by EF1A overexpression (Figure 4B).

To investigate whether the MERS-CoV N protein affects EF1A-mediated actin polymerization, actin polymers were analyzed using an *in vitro* actin polymerization assay followed by negative staining and electron microscopy. As shown in Figure 4C, slim and evenly distributed filaments of normal thickness were observed when globular actin (G-actin; monomeric) with EF1A or jasplakinolide, an actin linker agent, was used as a positive control (Figure 4C). In contrast, superbundled and disordered fibers with an increased diameter (12 vs. 25 nm) formed when G-actin was mixed with EF1A in the presence of the MERS-CoV N protein (Figure 4C). No fibers were observed in the negative control samples with G-actin alone or the cell-permeable actin polymerization inhibitor latrunculin B (Figure 4C).

Actin filament formation was further analyzed by *in vitro* high-speed co-sedimentation (lower panel of Figure 4C). High-speed centrifugation precipitated only bundled F-actin (polymeric); G-actin monomers remained in the supernatant. The results showed that the MERS-CoV N protein promoted G-actin polymerization *in vitro* (3rd lower panel of Figure 4C).

**FIGURE 5:**
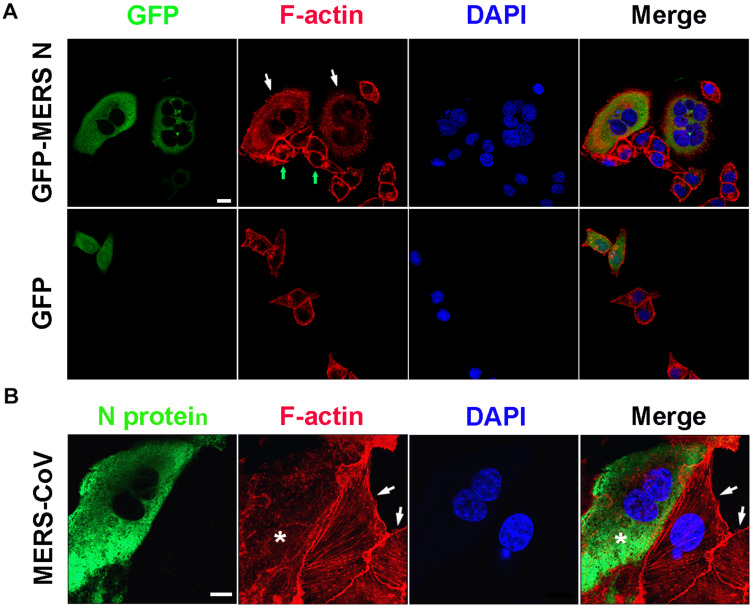
Actin bundling is disturbed in cells expressing the MERS-CoV N protein or infected with MERS-CoV. **(A)** HeLa cells transfected with the indicated plasmids were fixed and stained for immunofluorescence. Arrow: cells expressing GFP-MERS-CoV N or GFP only. Scale bar, 10 μm. **(B)** Vero E6 cells were infected with MERS-CoV and imaged by confocal microscopy. Green: MERS-CoV N stained with an anti-MERS-CoV N antibody and a FITC-labeled goat anti-mouse antibody. Red: F-actin stained with TRITC-phalloidin. Blue: nuclei stained with DAPI. Star: infected cell. Arrow: uninfected cell. Scale bar, 10 μm.

Consistent with the observation described above, obviously destroyed F-actin fibers assembly, dispersed distribution of actin without regular architecture and orientation, and thereby multiple nuclei were observed in HeLa cells expressing the GFP-MERS-CoV N protein (the white arrows), but not in the N protein-negative cells (the green arrows) in the same field of view or in the cells expressing GFP (Figure 5A). Similar abnormal F-actin fibers and multiple nuclei were also observed in Vero E6 cells infected with MERS-CoV (Figure 5B). Uninfected Vero E6 cells (without FITC signals) in the same observation field demonstrated normal F-actin fiber structures and numbers of nuclei (Figure 5B, arrowhead). Taken together, these findings suggest that the MERS-CoV N protein may bind to EF1A carried by G-actin, ultimately inducing the formation of abnormal actin bundles.

### The C-Terminal Region of the MERS-CoV N Protein Inhibits Cytokinesis

Elongation factor 1A regulates cytoskeletal organization, including the formation of contractile rings, which are pivotal cellular structures formed during cytokinesis and contain EF1A, p85, profilin, fimbrin, myosin, actin, and other protein components (Gonda and Numata, 2002; Bunai et al., 2006). Considering that the MERS-CoV N protein can induce cellular F-filament disorder by competitively binding with EF1A and interfering with F-filament assembly, we hypothesized that contractile ring-mediated cytokinesis might be affected by the MERS-CoV N protein, which in turn likely induces the formation of multinucleated cells.

**FIGURE 6:**
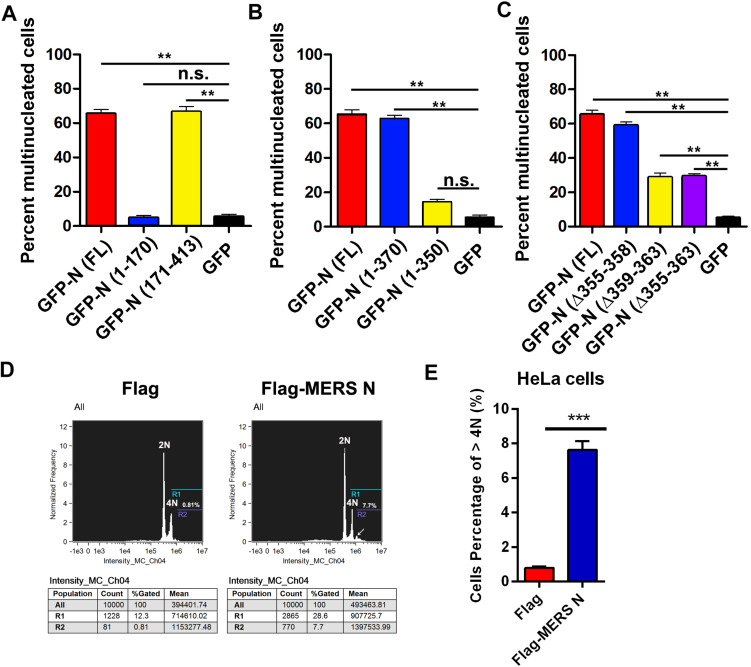
Residues 359–363 of the MERS-CoV N protein is the crucial motif involved in the N-induced multinucleation. **(A–C)** HeLa cells transfected with plasmids expressing the indicated proteins [GFP-N(FL) or deletion mutants] were fixed and stained for immunofluorescence. To calculate the ratio of multinucleated cells, at least 100 cells were counted for each sample. The data are presented as the mean ± SD of three independent experiments. **P < 0.01 vs. GFP by unpaired two-tailed Student’s *t*-test. **(D)** HeLa cells were transfected with Flag-vector or Flag-MERS N for 60 h. Then, the cytokinesis of HeLa cells were monitored by flow cytometry analysis after DNA staining with propidium iodide. **(E)** Quantitative flow cytometry data (cells percentage of >4N) are plotted in the graph. ***P < 0.001 vs. Flag by unpaired two-tailed Student’s *t*-test.

To verify this hypothesis, plasmids expressing GFP-tagged or mutant MERS-CoV N were transfected into HeLa cells. The cells were stained, and the ratios of multinucleated cells were determined by confocal microscopy. The ratio of multinucleated cells among cells expressing the full-length MERS-CoV N or the mutant N(171–413) was 13-fold higher than the ratio among cells expressing GFP or N(1–170), an N truncation mutant deficient in EF1A-binding activity (68 vs. 5%) (Figure 6A and Supplementary Figure 3A). Furthermore, the multinucleated cell ratio among cells expressing N(1–370) was fourfold higher than that among cells expressing N(1–350), an N truncation mutant that cannot interact with EF1A (65 vs. 15%) (Figure 6B and Supplementary Figure 3B). Consistent with the finding that amino acids 359–363 form the crucial motif involved in the N protein-EF1A interaction, significantly fewer multinucleated cells were observed among cells expressing N(Δ355–363) and N(Δ359–363) (Figure 6C and Supplementary Figure 3C) than among cells expressing the wild-type N protein. The ratio of multinucleated cells among cells expressing N(Δ355–358), a truncation mutant that can interact with EF1A (Figure 2C), was equal to that among cells expressing the wild-type N protein (Figure 6C). In addition, flow cytometry experiments further confirmed that MERS N leads to the formation of multinuclear cells (7.7 vs. 0.81%) by cytokinesis inhibition (Figures 6D,E). These findings collectively demonstrate that the MERS-CoV N protein can inhibit cytokinesis, thereby inducing multinucleation by disturbing EF1A-associated F-actin bundling. The C-terminal motif (residues 359–363) of the MERS-CoV N protein that is indispensable for EF1A association is at least partially responsible for MERS-CoV N-mediated cytokinesis inhibition.

### The MERS-CoV N Protein Promotes Protein Synthesis

**FIGURE 7:**
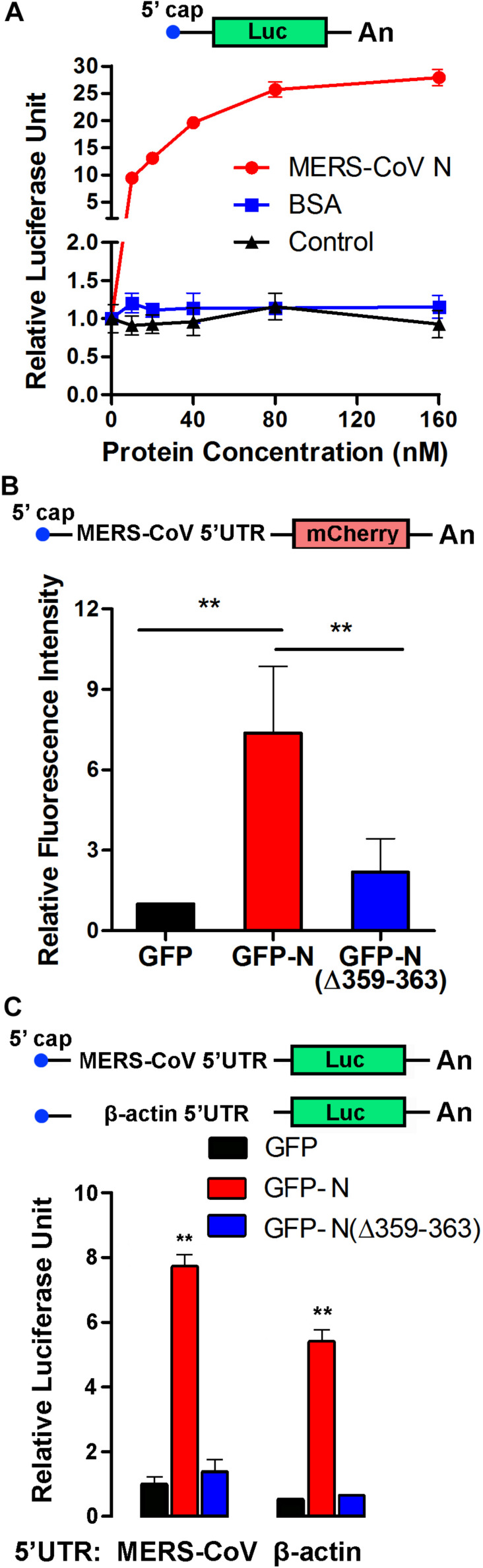
The MERS-CoV N protein promotes protein synthesis. **(A)** An *in vitro* translation assay for luciferase-encoding mRNA was performed using rabbit reticulocyte lysate system. Different concentrations of purified MERS-CoV N protein, BSA, or negative control of purified N protein (equal volumes) were added to the preincubation mixtures. Luciferase activity was measured with a luminometer. **(B)** The 5′UTR sequence was synthesized according to the sg mRNAs of MERS-CoV and inserted upstream of an mCherry reporter gene driven by the T7 promoter. Capped and polyadenylated RNA transcripts were synthesized using an mMESSAGE mMACHINE T7 Ultra Kit. HEK293T cells were transfected with reporter RNA 12 h after being transfected with plasmids expressing GFP-MERS-CoV N, GFP-MERS-CoV N(Δ359–363), or GFP. The level of mCherry expression was detected 30 h later using a multilabel plate reader. The data are presented as the mean ± SD of three independent experiments. **P < 0.01 by unpaired two-tailed Student’s *t*-test. **(C)** The 5′UTR sequence was synthesized according to the sg mRNAs of MERS-CoV or the mRNA of β-actin and inserted upstream of a luciferase reporter gene driven by the T7 promoter. The cell transfection details and experimental statistics are the same as in panel **(B)**.

Elongation factor 1A is necessary for the elongation of translation and broadly contributes to the virus life cycle. To investigate the effect of the MERS-CoV N protein on translation, an *in vitro* translation assay was performed. The data showed that the purified MERS-CoV N protein dose-dependently increased luciferase expression up to 30-fold (Figure 7A). To determine the effect of the MERS-CoV N protein on protein translation in cells, a MERS-CoV-transcript-like mCherry-encoding reporter RNA carrying a CoV 5′UTR that was designed according the MERS-CoV sequence was constructed (Figure 7B). HEK293T cells were first transfected with pEGFP-C1-N, pEGFP-C1-N(Δ359–363), or pEGFP-C1 vectors. After 24 h, the cells were transfected with mCherry reporter RNA. The level of mCherry was quantitatively analyzed by a fluorospectrophotometer. The results showed that GFP-N significantly promoted mCherry translation, resulting in translation levels up to ∼7-fold higher than those mediated by the GFP-only control (Figure 7B). Moreover, compared to the N protein, N protein (NΔ359–363) showed a greatly compromised induction on the expression of mCherry (∼2-fold compared to GFP) (Figure 7B). These results suggest that EF1A-involved mRNA translation is regulated by the N protein of MERS-CoV, although it could not exclude the possibility that the N protein promoted translation by regulating other components of translation.

To determine whether the effect of the MERS-CoV N protein on translation is specific to MERS-CoV, reporter RNAs encoding luciferase and carrying the 5′UTR of sg mRNA from MERS-CoV or the 5′UTR of β-actin mRNA were constructed (Figure 7C). HEK293T cells were pretransfected with plasmids encoding GFP-MERS-CoV N (GFP-N), GFP-MERS-CoV N(Δ359–363) [GFP-N(Δ359–363)], or GFP (as a control). Twelve hours later, the cells were transfected with the luciferase reporter RNAs, and the translation activity was detected after 24 h. As shown in Figure 7C, the amount of luciferase translated from the MERS-CoV-transcript-like reporter RNA was eightfold higher in the presence of MERS-CoV N than in the presence of GFP. The truncated MERS-CoV N protein GFP-N(Δ359–363) had little, if any, effect on luciferase translation. Notably, the MERS-CoV N protein similarly regulated the translation of luciferase from the RNA construct carrying the 5′UTR of β-actin mRNA (Figure 7C), indicating that the MERS-CoV N protein can generally promote RNA translation activity in host cells and that this effect is not limited to MERS-CoV-specific transcripts.

## Discussion

The N proteins of coronaviruses enhance the efficiency of viral transcription, translation, and assembly (Baric et al., 1988; Stohlman et al., 1988; McBride et al., 2014). The N protein of MHV binds not only to the positive-sense of genomic-length RNA but also to all six sg mRNAs (Baric et al., 1988; Stohlman et al., 1988), and has been found to stimulate translation of a reporter mRNA carrying the MHV 5′UTR (Tahara et al., 1998). Several reports have shown that proteins from coronaviruses play roles at the translational initiation step (Nakagawa et al., 2016); nevertheless, little is known about whether these viruses affect elongation. Our data showed that the translation activity of EF1A was promoted by the MERS-CoV N protein (Figure 7), and this promotion may contribute to viral protein synthesis. This finding is different from that of a previous study revealing an inhibitory effect of the SARS-CoV N protein on EF1A translation activity (Zhou et al., 2008) and updates the existing knowledge regarding the role of the MERS-CoV N protein in MERS-CoV infected cells.

Elongation factor 1A plays a critical role in replication in several viruses and is routinely exploited by viral proteins. EF1A is part of the HIV reverse transcription complex and co-immunoprecipitates with the p51 subunit of reverse transcriptase (Warren et al., 2012). EF1A also facilitates viral replication complex assembly and favors replication of West Nile virus and dengue virus (Davis et al., 2007; Abbas et al., 2015). Furthermore, EF1A interacts with the N protein of TGEV and favors TGEV replication (Zhang et al., 2014). EF1A has been observed in highly purified virions from numerous RNA and DNA viruses, including SARS-CoV, cytomegalovirus, HIV-1, vesicular stomatitis virus, and vaccinia virus, among others (Abbas et al., 2015), suggesting the critical role of EF1A in the viral life cycle. Viral hijacking of EF1A may affect its intrinsic functions.

In addition to playing a role in protein translation, EF1A, a pleiotropic protein, is involved in actin bundling in cells (Kurasawa et al., 1996; Abbas et al., 2015). During cytokinesis, EF1A localizes to the division furrow instead of being diffusely distributed in the cytoplasm, and it is involved in the formation of F-actin bundles in the contractile ring (Kurasawa et al., 1996; Numata et al., 2000). There is evidence that the proteins of some viruses interact with EF1A to block F-actin bundling. For example, the interaction of HBx of hepatitis B virus (HBV) with EF1A results in blockade of actin bundling (Lin et al., 2012). In addition, the E7 protein of cutaneous human papilloma virus type 38 (HPV38) has been shown to directly interact with EF1A and induce actin stress fiber disruption (Yue et al., 2011). The findings of our study confirm the interaction of EF1A with MERS-CoV N and reveal that this interaction blocks the association between EF1A and actin bundling; in turn, this effect causes cytokinesis blockade in cells infected with MERS-CoV, resulting in abnormal mitosis and multiple nuclei formation.

Direct and indirect interactions between viral nucleocapsids and actin have been widely documented and are generally used for intracellular transport of nucleocapsids, which is crucial for viral infection or packaging (van Loo et al., 2001; Xu et al., 2007; Schudt et al., 2013, 2015). In this study, abnormal F-actin fibers were observed in cells with N protein expression mediated by plasmid transfection and MERS-CoV infection. Disordered inactive F-actin fibers were also detected in an *in vitro* actin polymerization assay in the presence of the MERS-CoV N protein. This study provides the first evidence that the MERS-CoV N protein induces disordered F-actin fiber formation. However, further studies are needed to reveal whether abnormal F-actin fibers are essential for steps in the viral life cycle such as nucleocapsid transport and virion packaging.

## Data Availability Statement

The original contributions presented in the study are included in the article/Supplementary Material, further inquiries can be directed to the corresponding author/s.

## Author Contributions

CC and XL designed the experiments. LZ, TG, and YF performed the experiments and wrote the manuscript. WY, YL, JY, XH, QD, HL, and YH analyzed the data. YJ, PL, CC, and XL reviewed the manuscript. All authors contributed to the article and approved the submitted version.

## Conflict of Interest

The authors declare that the research was conducted in the absence of any commercial or financial relationships that could be construed as a potential conflict of interest.

## References

[B1] Abbas W. Kumar A. Herbein G. (2015). The eef1a proteins: at the crossroads of oncogenesis, apoptosis, and viral infections. *Front Oncol.* 5:75. 10.3389/fonc.2015.00075 25905039 PMC4387925

[B2] Arabi Y. M. Harthi A. Hussein J. Bouchama A. Johani S. Hajeer A. H. (2015). Severe neurologic syndrome associated with Middle East respiratory syndrome corona virus (MERS-CoV). *Infection* 43 495–501. 10.1007/s15010-015-0720-y 25600929 PMC4521086

[B3] Baric R. S. Nelson G. W. Fleming J. O. Deans R. J. Keck J. G. Casteel N. (1988). Interactions between coronavirus nucleocapsid protein and viral RNAs: implications for viral transcription. *J. Virol.* 62 4280–4287. 10.1128/JVI.62.11.4280-4287 2845140 PMC253862

[B4] Bunai F. Ando K. Ueno H. Numata O. (2006). Tetrahymena eukaryotic translation elongation factor 1A (eEF1A) bundles filamentous actin through dimer formation. *J. Biochem.* 140 393–399. 10.1093/jb/mvj169 16877446

[B5] Cimarelli A. Luban J. (1999). Translation elongation factor 1-alpha interacts specifically with the human immunodeficiency virus type 1 Gag polyprotein. *J. Virol.* 73 5388–5401. 10364286 10.1128/jvi.73.7.5388-5401.1999 PMC112595

[B6] Davis W. G. Blackwell J. L. Shi P. Y. Brinton M. A. (2007). Interaction between the cellular protein eEF1A and the 3’-terminal stem-loop of West Nile virus genomic RNA facilitates viral minus-strand RNA synthesis. *J. Virol.* 81 10172–10187. 10.1128/JVI.00531-07 17626087 PMC2045417

[B7] Ejiri S. (2002). Moonlighting functions of polypeptide elongation factor 1: from actin bundling to zinc finger protein R1-associated nuclear localization. *Biosci. Biotechnol. Biochem.* 66 1–21. 10.1271/bbb.66.1 11866090

[B8] Fessenden J. M. Cairncross J. Moldave K. (1963). Studies on polynucleotide-stimulated amino acyl transfer from soluble-RNA to rat liver ribosomes. *Proc. Natl. Acad. Sci. U.S.A.* 49 82–88. 10.1128/JVI.62.11.4280-4287 13944965 PMC300632

[B9] Gonda K. Numata O. (2002). p85 binds to G-actin in a Ca(2+)/calmodulin-dependent manner, thus regulating the initiation of cytokinesis in tetrahymena. *Biochem. Biophys. Res. Commun.* 292 1098–1103. 10.1006/bbrc.2002.6777 11944929

[B10] Gross S. R. Kinzy T. G. (2005). Translation elongation factor 1A is essential for regulation of the actin cytoskeleton and cell morphology. *Nat. Struct. Mol. Biol.* 12 772–778. 10.1038/nsmb979 16116436

[B11] Hasler J. Rada C. Neuberger M. S. (2011). Cytoplasmic activation-induced cytidine deaminase (AID) exists in stoichiometric complex with translation elongation factor 1 alpha (eEF1A). *Proc. Natl. Acad. Sci. U.S.A.* 108 18366–18371. 10.1073/pnas.1106729108 22042842 PMC3215039

[B12] Ksiazek T. G. Erdman D. Goldsmith C. S. Zaki S. R. Peret T. Emery S. (2003). A novel coronavirus associated with severe acute respiratory syndrome. *N. Engl. J. Med.* 348 1953–1966. 10.1056/NEJMoa030781 12690092

[B13] Kurasawa Y. Hanyu K. Watanabe Y. Numata O. (1996). F-actin bundling activity of Tetrahymena elongation factor l is regulated by Ca2+/calmodulin. *J. Biochem.* 119 791–798. 10.1093/oxfordjournals.jbchem.a021309 8743583

[B14] Lai M. M. Cavanagh D. (1997). The molecular biology of coronaviruses. *Adv. Virus Res.* 48 1–100. 10.1016/S0065-3527(06)66005-3 9233431 PMC7130985

[B15] Li F. Q. Tam J. P. Liu D. X. (2007). Cell cycle arrest and apoptosis induced by the coronavirus infectious bronchitis virus in the absence of p53. *Virology* 365 435–445. 10.1016/j.virol.2007.04.015 17493653 PMC7103336

[B16] Li Y.-H. Hu C.-Y. Wu N.-P. Yao H.-P. Li L.-J. (2019). Molecular characteristics, functions, and related pathogenicity of MERS-CoV proteins. *Engineering* 5 940–947. 10.1016/j.eng.2018.11.035 32288963 PMC7104727

[B17] Lin W. S. Jiao B. Y. Wu Y. L. Chen W. N. Lin X. (2012). Hepatitis B virus X protein blocks filamentous actin bundles by interaction with eukaryotic translation elongat ion factor 1 alpha 1. *J. Med. Virol.* 84 871–877. 10.1002/jmv.23283 22499008

[B18] McBride R. van Zyl M. Fielding B. C. (2014). The coronavirus nucleocapsid is a multifunctional protein. *Viruses* 6 2991–3018. 10.3390/v6082991 25105276 PMC4147684

[B19] Nakagawa K. Lokugamage K. G. Makino S. (2016). Viral and cellular mRNA translation in coronavirus-infected cells. *Adv. Virus Res.* 96 165–192. 10.1016/bs.aivir.2016.08.001 27712623 PMC5388242

[B20] Nguyen T. H. V. Lichiere J. Canard B. Papageorgiou N. Attoumani S. Ferron F. (2019). Structure and oligomerization state of the C-terminal region of the Middle East respiratory syndrome coronavirus nucleoprotein. *Acta Crystallogr. D Struct. Biol.* 75 8–15. 10.1107/S2059798318014948 30644840 PMC7159728

[B21] Numata O. Kurasawa Y. Gonda K. Watanabe Y. (2000). Tetrahymena elongation factor-1 alpha is localized with calmodulin in the division furrow. *J. Biochem.* 127 51–56. 10.1093/oxfordjournals.jbchem.a022583 10731666

[B22] Papageorgiou N. Lichière J. Baklouti A. Ferron F. Sevajol M. Canard B. (2016). Structural characterization of the N-terminal part of the MERS-CoV nucleocapsid by X-ray diffraction and small-angle X-ray scatteringarticle. *Acta Crystallogr. D Struct. Biol.* 72 192–202. 10.1107/s2059798315024328 26894667 PMC7159594

[B23] Peiris J. S. Guan Y. Yuen K. Y. (2004). Severe acute respiratory syndrome. *Nat. Med.* 10 S88–S97. 10.1038/nm1143 15577937 PMC7096017

[B24] Schudt G. Dolnik O. Kolesnikova L. Biedenkopf N. Herwig A. Becker S. (2015). Transport of ebolavirus nucleocapsids is dependent on actin polymerization: live-cell imaging analysis of ebolavirus-infected cells. *J. Infect. Dis.* 212 S160–S166. 10.1093/infdis/jiv083 26038396

[B25] Schudt G. Kolesnikova L. Dolnik O. Sodeik B. Becker S. (2013). Live-cell imaging of Marburg virus-infected cells uncovers actin-dependent transport of nucleocapsids over long distances. *Proc. Natl. Acad. Sci. U.S.A.* 110 14402–14407. 10.1073/pnas.1307681110 23940347 PMC3761630

[B26] Stohlman S. A. Baric R. S. Nelson G. N. Soe L. H. Welter L. M. Deans R. J. (1988). Specific interaction between coronavirus leader RNA and nucleocapsid protein. *J. Virol.* 62 4288–4295. 10.1128/JVI.62.11.4288-4295.1988 2845141 PMC253863

[B27] Surjit M. Liu B. P. Jameel S. Chow V. T. K. Lal S. K. (2004). The SARS coronavirus nucleocapsid protein induces actin reorganization and apoptosis in COS-1 cells in the absence of growth factors. *Biochem. J.* 383 13–18. 10.1042/bj20040984 15294014 PMC1134038

[B28] Tahara S. M. Dietlin T. A. Nelson G. W. Stohlman S. A. Manno D. J. (1998). Mouse hepatitis virus nucleocapsid protein as a translational effector of viral mRNAs. *Adv. Exp. Med. Biol.* 440 313–318. 10.1007/978-1-4615-5331-1_41 9782298

[B29] van Loo N. D. Fortunati E. Ehlert E. Rabelink M. Grosveld F. Scholte B. J. (2001). Baculovirus infection of nondividing mammalian cells: mechanisms of entry and nuclear transport of capsids. *J. Virol.* 75 961–970. 10.1128/JVI.75.2.961-970.2001 11134309 PMC113992

[B30] Warren K. Wei T. Li D. Qin F. Warrilow D. Lin M. H. (2012). Eukaryotic elongation factor 1 complex subunits are critical HIV-1 reverse transcription cofactors. *Proc. Natl. Acad. Sci. U.S.A.* 109 9587–9592. 10.1073/pnas.1204673109 22628567 PMC3386134

[B31] Xu H. Yao L. Lu S. Qi Y. (2007). Host filamentous actin is associated with *Heliothis armigera* single nucleopolyhedrosis virus (HaSNPV) nucleocapsid transport to the host nucleus. *Curr. Microbiol.* 54 199–206. 10.1007/s00284-006-8261-3 17294324

[B32] Xu Z. Shi L. Wang Y. Zhang J. Huang L. Zhang C. (2020). Pathological findings of COVID-19 associated with acute respiratory distress syndrome. *Lancet Respir. Med.* 8 420–422. 10.1016/S2213-2600(20)30076-X 32085846 PMC7164771

[B33] Yang C. W. Lee Y. Z. Kang I. J. Barnard D. L. Jan J. T. Lin D. (2010). Identification of phenanthroindolizines and phenanthroquinolizidines as novel potent anti-coronaviral agents for porcine enteropathogenic coronavirus transmissible gastroenteritis virus and human severe acute respiratory syndrome coronavirus. *Antiviral Res.* 88 160–168. 10.1016/j.antiviral.2010.08.009 20727913 PMC7114283

[B34] Yue J. Shukla R. Accardi R. Zanella-Cleon I. Siouda M. Cros M. P. (2011). Cutaneous human papillomavirus type 38 E7 regulates actin cytoskeleton structure for increasing cell proliferation through CK2 and the eukaryotic elongation factor 1A. *J. Virol.* 85 8477–8494. 10.1128/JVI.02561-10 21697493 PMC3165781

[B35] Zaki A. M. van Boheemen S. Bestebroer T. M. Osterhaus A. D. Fouchier R. A. (2012). Isolation of a novel coronavirus from a man with pneumonia in Saudi Arabia. *N. Engl. J. Med.* 367 1814–1820. 10.1056/NEJMoa1211721 23075143

[B36] Zhang X. Shi H. Chen J. Shi D. Li C. Feng L. (2014). EF1A interacting with nucleocapsid protein of transmissible gastroenteritis coronavirus and plays a role in virus replication. *Vet. Microbiol.* 172 443–448. 10.1016/j.vetmic.2014.05.034 24974120 PMC7117464

[B37] Zhou B. Liu J. Wang Q. Liu X. Li X. Li P. (2008). The nucleocapsid protein of severe acute respiratory syndrome coronavirus inhibits cell cytokinesis and proliferation by interacting with translation elongation factor 1alpha. *J. Virol.* 82 6962–6971. 10.1128/JVI.00133-08 18448518 PMC2446950

[B38] Zhou J. Chu H. Chan J. F. Yuen K. Y. (2015). Middle East respiratory syndrome coronavirus infection: virus-host cell interactions and implications on pathogenesis. *Virol. J.* 12:218. 10.1186/s12985-015-0446-6 26690369 PMC4687146

