# Supplementary material for: The MERS-CoV N Protein Regulates Host Cytokinesis and Protein Translation via Interaction With EF1A
Source: Front Microbiol. 2021 Jun 23;12:551602. doi: 10.3389/fmicb.2021.551602 (PMC8261062; doi:10.3389/fmicb.2021.551602)
Supplement: Supplementary file 1 [file Data_Sheet_1.docx]

Supplementary Material


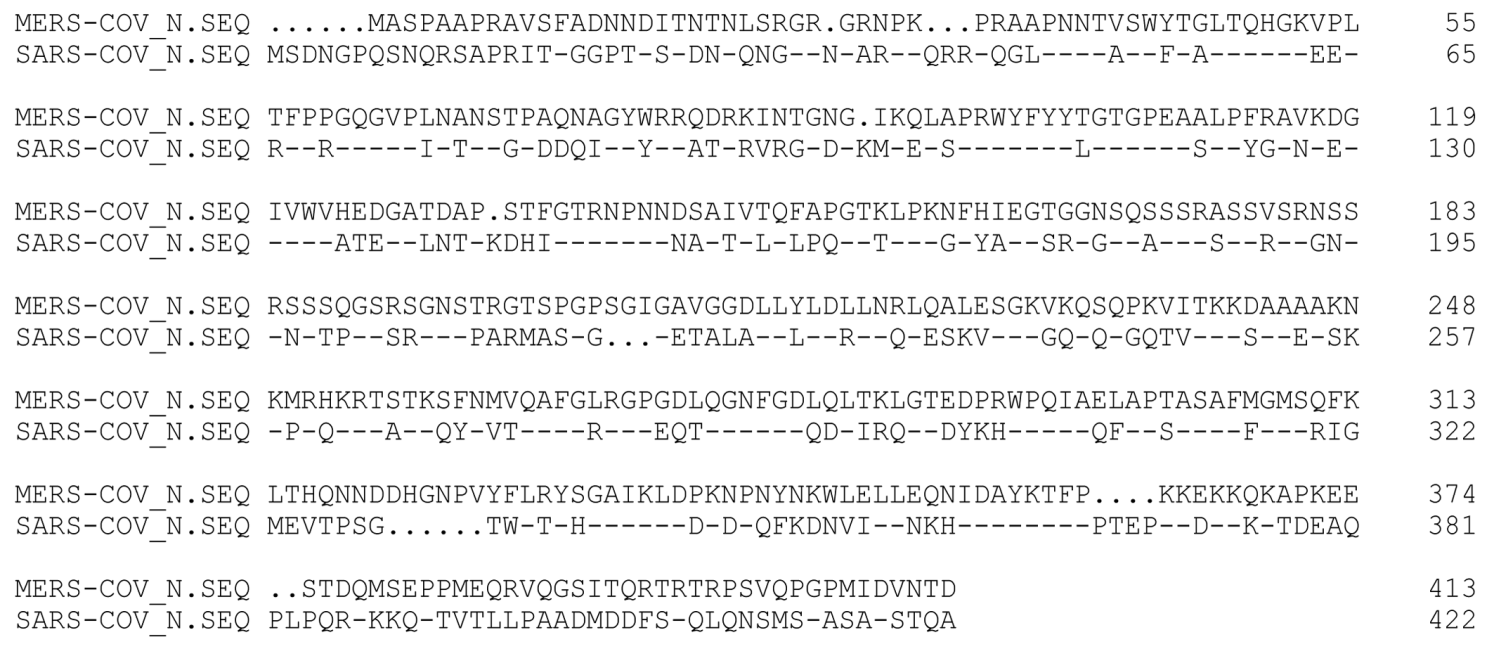


Supplementary Figure S1. Comparison of the protein sequences of the SARS-CoV N protein and the MERS-CoV N protein. Sequence alignment was done using DNAMAN software. Dot indicate gaps in sequence alignment. Dashes indicate fully identical amino acids.

**
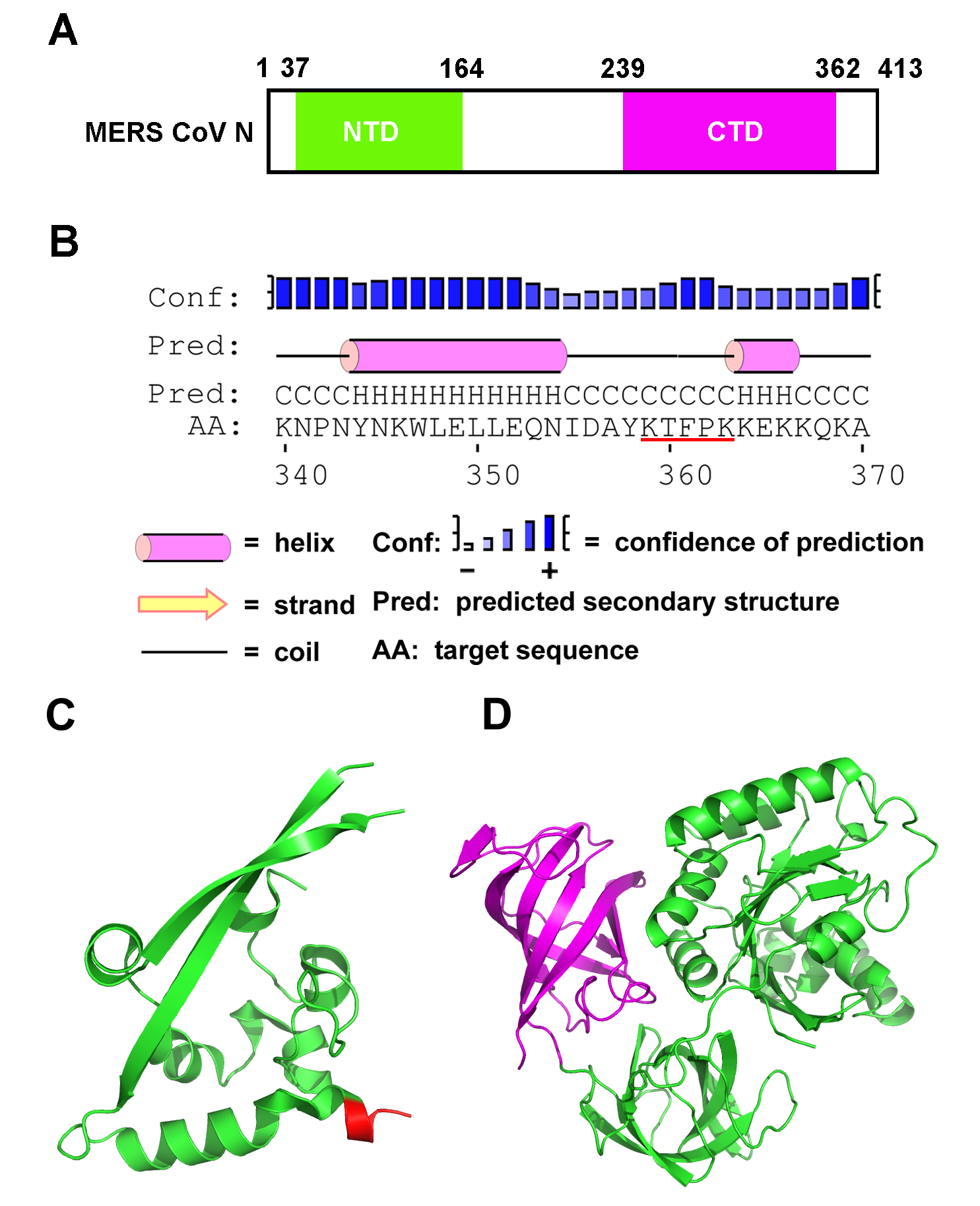
**

**Supplementary Figure S2.** Schematic diagram of the MERS-CoV N protein domain. (**A**) Functional domain of the MERS-CoV N protein. (**B**) The secondary structure elements of amino acid residues 340-370 of the MERS-CoV N protein were predicted based on an ESPript algorithm. The underlined symbols indicate amino acid residues of 359-363. (**C**) The tertiary molecular structure illustrations of the CTD of the MERS-CoV N protein (PDB: 6G13). The amino acid residues 359-362 (KTFP) of the MERS-CoV N protein are shown in red, and the remaining amino acids are shown in green. (**D**) The tertiary molecular structure illustrations of the EF1A protein (PDB: 6ZMO CD Chains). The amino acid residues 336-462 of the EF1A protein are shown in purple, and the remaining amino acids are shown in green.


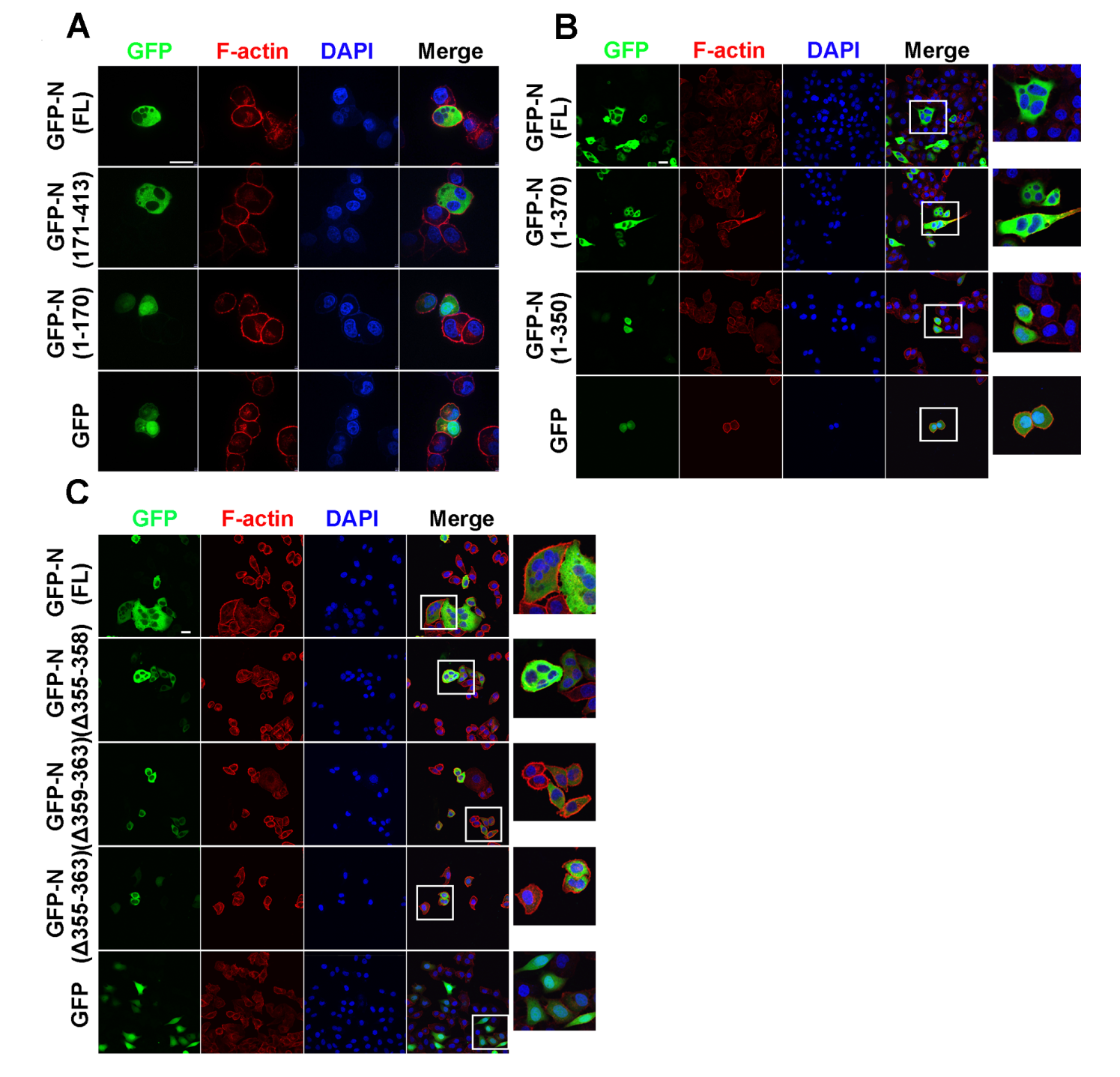


**Supplementary Figure S3.** Residues 359-363 of the MERS-CoV N protein is the crucial region in cytokinesis inhibition by promoting the formation of multinucleated cells. (**A**-**C**) HeLa cells transfected with plasmids expressing GFP-MERS-CoV N (FL) or deletion mutants were fixed and stained for immunofluorescence. Green: GFP-MERS-CoV N, GFP-MERS-CoV N mutant, or GFP only. Red: F-actin stained with TRITC-labeled phalloidin. Blue: nuclei stained with DAPI. Scale bar, 20 μm.
